# An Intelligent Temperature Compensation Method for Pressure Sensors Under High-Temperature and High-Pressure Conditions Based on a Modified Slime Mold Algorithm

**DOI:** 10.3390/mi17040398

**Published:** 2026-03-25

**Authors:** Yang Zhao, Wanlu Jiang, Enyu Tang, Chengpeng Yu, Mengda Zhang, Zhenbao Li, Yongyong Li

**Affiliations:** 1 School of Mechanical Engineering, Yanshan University, Qinhuangdao 066004, China; 2 Hebei Provincial Key Laboratory of Heavy Machinery Fluid Power Transmission and Control, Yanshan University, Qinhuangdao 066004, China; 3 Engineering Technology Research Institute of CNPC Xibu Drilling Engineering Company Limited, Karamay 834000, China

**Keywords:** temperature compensation, Modified Slime Mold Algorithm (MSMA), embedded system implementation, piezoresistive pressure sensor

## Abstract

During deep and ultra-deep oil and gas drilling, downhole high-temperature and high-pressure conditions significantly affect the measurement accuracy of piezoresistive pressure sensors. To improve measurement accuracy under such extreme conditions, this study proposes an intelligent temperature compensation method based on a Modified Slime Mold Algorithm (MSMA). An experimental platform covering the full operating range of 0–175 °C and 0–170 MPa was established to acquire sensor outputs, and samples were collected at various temperature and pressure points to construct a dataset. Key parameters of the compensation model were optimized using the MSMA, enhancing the model’s fitting capability. Results indicate that, after compensation, the sensor exhibits a maximum full-scale error of 0.26% and a maximum sensitivity drift of −0.019% FS/°C, significantly reducing errors compared with traditional interpolation and polynomial fitting methods. The optimized compensation model was further deployed on an embedded hardware platform, enabling high-precision temperature compensation in an engineering context. Experimental data demonstrate that the embedded implementation maintains compensation accuracy while meeting real-time application requirements, making it suitable for downhole pressure monitoring and for output correction of other intelligent sensors operating under complex field conditions.

## 1. Introduction

Oil and natural gas, as vital constituents of the global energy system, maintain their irreplaceable significance despite the ongoing global energy transition. Energy security is essential for national stability and development, directly influencing both economic growth and technological progress. As conventional oil and gas resources are increasingly depleted globally, the exploration and development of unconventional oil and gas resources have become key trends in the evolution of the global energy industry [1]. In this context, deep and ultra-deep well exploration and production have emerged as effective approaches to enhancing oil and gas production efficiency. Under the extreme high-temperature and high-pressure conditions of ultra-deep wells, accurate monitoring of formation and engineering parameters is essential for improving drilling efficiency and ensuring operational safety [2]. In ultra-deep well environments, downhole temperatures may reach up to 175 °C, and pressures can rise to 170 MPa, accompanied by severe vibrations and dynamic temperature fluctuations. Parameters such as annular pressure, differential pressure, and weight on bit not only directly influence well control safety, borehole stability, and drill string operating conditions but also play critical roles in formation identification and real-time decision-making [3]. Therefore, acquiring reliable downhole pressure data under extreme high-temperature and high-pressure conditions is of great significance for enhancing drilling efficiency and ensuring operational safety.

Under the limited installation space and harsh downhole conditions, micro-electro-mechanical systems (MEMS) piezoresistive sensors are widely used in drilling engineering owing to their compact structure, high accuracy, and broad environmental adaptability [4]. However, the sensing elements of MEMS piezoresistive pressure sensors are primarily composed of silicon-based materials, whose piezoresistive coefficients, resistance values, and diaphragm mechanical properties vary significantly with temperature. High temperatures cause pronounced sensitivity drift and zero-offset drift, and also induce complex errors such as enhanced diaphragm nonlinearity. High-pressure conditions further intensify stress coupling within the sensing diaphragm, making the temperature–pressure nonlinear relationship even more intricate. These factors collectively result in a pronounced degradation of sensor output accuracy under ultra-deep well conditions, which has become a critical bottleneck limiting the reliability of downhole measurements [5]. Therefore, investigating and implementing high-precision compensation methods to address the substantial errors exhibited by piezoresistive pressure sensors in high-temperature and high-pressure environments has become one of the key research directions in current downhole measurement technology.

At present, compensation methods for downhole pressure sensors can be categorized into hardware and software approaches [6]. Hardware compensation mainly corrects the sensor output through circuit design, material selection, and structural optimization. Developing novel sensors with improved measurement accuracy and stability offers a solution to the challenges of downhole measurements, and exploring alternatives with inherently higher stability based on sensing principles is also a key pathway to overcoming the bottlenecks of sensing in extreme environments. In the field of piezoelectric sensing for extreme environments, Kim et al. achieved flexible high-quality single-crystal thin films and advanced the working temperature of AlN films to 900 °C [7,8], and, more recently, widely used application-specific integrated circuits (ASICs). Passive compensation circuits rely on bridge balancing resistors and thermistors (NTC/PTC) to counteract sensor thermal drift through the temperature characteristics of these components. With advances in integrated circuit technology, some commercial sensors (e.g., Kulite, Endevco, Bosch, and Gefran) employ ASIC-based digital compensation, which uses on-chip ADCs, temperature sensors, and lookup-table or polynomial algorithms to achieve temperature compensation. Although ASIC solutions offer high compensation accuracy and good stability within conventional industrial temperature ranges, their compensation models are relatively fixed and ill-suited for the highly coupled, rapidly varying temperature–pressure conditions encountered in ultra-deep drilling. Additionally, once packaged, ASICs cannot be updated or recalibrated, and their manufacturing costs are high, limiting their applicability to specific high-precision scenarios [9]. Consequently, software compensation has become the mainstream approach for piezoresistive pressure sensors.

Software compensation corrects sensor drift and nonlinearities through data processing algorithms applied to the sensor output. According to methodological differences, software compensation can be divided into parametric and nonparametric approaches. Parametric methods rely on explicit mathematical models that establish functional relationships between environmental variables (e.g., temperature and pressure) and sensor errors for correction, typically using look-up table, interpolation, or surface fitting techniques [10,11,12]. However, parametric methods have notable limitations. The lookup-table method fundamentally depends on predefined discrete calibration points. Tables cannot fully cover the entire operating domain, and increasing their density sharply increases memory requirements. Moreover, because no continuous mathematical model exists between grid points, the compensation performance becomes highly sensitive to the distribution of calibration points, making it difficult to maintain accuracy and stability in unseen regions. Interpolation methods depend on the local distribution of surrounding calibration points; when these points are sparse or unevenly distributed, significant local interpolation errors may occur. High-order interpolation further increases the risk of oscillations during computation, making the compensation outcome sensitive to small data perturbations. Additionally, interpolation struggles to capture complex nonlinear temperature–pressure coupling relationships, limiting its stability and generalization capability. Polynomial fitting suffers from the rapid growth of the coefficient matrix condition number as the polynomial order increases, making the model highly sensitive to small data perturbations and destabilizing the coefficient estimation process, which compromises the reliability of the compensation model. Surface fitting involves numerous parameters and high computational complexity, making it susceptible to data noise and gaps in calibration coverage; increasing the fitting order similarly leads to instability and heightened sensitivity to data perturbations.

Nonparametric compensation, built upon intelligent optimization algorithms and neural network theory, does not rely on explicit functional models. Instead, it learns the error characteristics directly from sensor calibration data, enabling effective correction of complex and highly nonlinear errors. As a result, it offers stronger adaptability and higher compensation accuracy [13]. The nonlinear approximation capability of neural networks enables them to learn underlying mapping relationships from large volumes of complex data, making them well-suited for multivariable and strongly nonlinear systems that traditional methods struggle to handle. In pressure sensor output compensation, neural networks do not rely on explicit functional forms, allowing for more stable and higher-accuracy error correction. Through forward propagation and error back-propagation, neural networks iteratively adjust their weights and biases based on training data, thereby compensating for the sensor’s output characteristics. As the size of the training dataset increases and the network architecture is further optimized, the model’s generalization capability and compensation accuracy can be further enhanced. Studies [14,15,16] have applied backpropagation (BP) neural networks, extreme learning machines (ELMs), and least-squares support vector machines (LSSVMs) to compensate for temperature-induced nonlinear errors in pressure sensors, and all reported metrics improved measurement accuracy. However, BP neural networks exhibit slow convergence and are prone to local optima; although ELM offers fast training, its performance is highly sensitive to the random initialization of input weights and biases, undermining model stability; the LSSVM, when dealing with high-dimensional feature spaces, requires carefully tuned hyperparameters, and its performance is strongly influenced by parameter selection due to the lack of a universal optimization mechanism. These limitations reduce model generalization capability and result in considerable variability and uncertainty in compensation performance under different operating conditions. To address the shortcomings of these methods in training efficiency, model stability, and generalization, studies [17,18,19] have employed metaheuristic optimization algorithms to optimize the weights and biases of neural networks, demonstrating their feasibility in sensor output compensation and neural network optimization. Nonetheless, these algorithms still face notable limitations when applied to complex nonlinear modeling tasks. Specifically, particle swarm optimization (PSO) relies heavily on historical best solutions during population updates, making it prone to premature convergence and insufficient global exploration in complex search spaces. The whale optimization algorithm (WOA), when handling high-dimensional optimization problems, often suffers from limited convergence accuracy and sensitivity to the initial population. The gravitational search algorithm (GSA), which is based on mass-attraction mechanisms, tends to exhibit population clustering in later iterations, resulting in degraded search capability and increased computational complexity, which negatively impacts optimization efficiency and stability. These limitations constrain the generalization and robustness of related models in dynamic environments and hinder their suitability for meeting the requirements of high-precision sensor measurement and low-cost deployment. Table 1 lists various compensation algorithms, including traditional methods such as lookup tables, interpolation, and curvilinear fitting, as well as advanced algorithms like neural networks (BP), LSSVM, and swarm optimization. These methods differ in terms of temperature range, pressure range, accuracy, and computational overhead. It is important to note that the calibration accuracy may vary even when the same algorithm is used, which is primarily due to differences in experimental components and environmental conditions [6,15,20,21].

Therefore, it is necessary to explore more efficient intelligent compensation methods with strong global optimization capability to improve the measurement accuracy of pressure sensors under complex operating conditions. For temperature-induced nonlinear errors in pressure sensors, hardware-based correction can mitigate thermal drift to some extent, but suffers from drawbacks such as structural complexity, high cost, and poor adaptability. Software-based compensation methods—particularly those incorporating neural networks—have shown strong potential for enhancing compensation accuracy. However, neural networks themselves are limited by issues such as susceptibility to local optima during training, sensitivity to initial weight settings, and unstable generalization performance, all of which restrict their practicality and broader application. Although existing studies have attempted to optimize neural network weights and biases through metaheuristic algorithms to improve modeling accuracy, these approaches generally encounter challenges in high-dimensional spaces, including premature convergence, low computational efficiency, and increased training complexity.

In response to these challenges, recent studies have explored the use of intelligent optimization algorithms to assist in neural network structure optimization and parameter initialization. Among them, the Slime Mould Algorithm (SMA) has attracted considerable attention in function optimization and parameter tuning due to its simple structure, strong global search capability, and fast convergence speed. SMA simulates the foraging behavior of slime mould to efficiently explore complex solution spaces, making it well-suited for optimizing neural network weights and biases, thereby avoiding local optima and enhancing overall network performance. However, similar to other swarm intelligence algorithms, the balance between global exploration and local exploitation largely determines its efficiency and effectiveness. It is therefore crucial to achieve a proper balance in SMA’s global and local search capabilities, enabling rapid global exploration while ensuring quick convergence to the global optimum [22]. Compared with traditional swarm intelligence algorithms such as particle swarm optimization and genetic algorithms, SMA maintains strong global exploration while achieving faster convergence and requiring fewer parameters, making it particularly suitable for the high-dimensional, nonlinear, and strongly coupled temperature compensation optimization tasks addressed in this study. Accordingly, this work adopts SMA and further proposes an improved version, MSMA, to enhance the output accuracy and stability of BP neural networks.

In this study, a BP neural network optimization method based on the Modified Slime Mold Algorithm (MSMA) is proposed. By introducing chaotic initialization, elite opposition-based learning, and an adaptive step-size strategy, the MSMA effectively balances global exploration and local exploitation, thereby enhancing the generalization capability of the proposed network in nonlinear compensation tasks. Furthermore, unlike conventional approaches that rely solely on pressure signals, the proposed model incorporates both temperature and pressure as dual inputs to capture the coupled influence of temperature on the sensor’s output characteristics, leading to improved accuracy and robustness in compensation performance.

The rest of this paper is organized as follows: Section 2 presents an analysis of the sensor performance parameters, theoretical background, and mechanisms underlying sensor error generation. Section 3 describes the optimization framework of the pro-posed Modified Slime Mold Algorithm (MSMA), the temperature compensation model, the experimental setup and the data acquisition process, as well as the corresponding compensation performance analysis. Finally, Section 4 concludes the paper.

## 2. Theoretical Background

### 2.1. Working Principle of Piezoresistive Pressure Sensors

**Figure 1:**
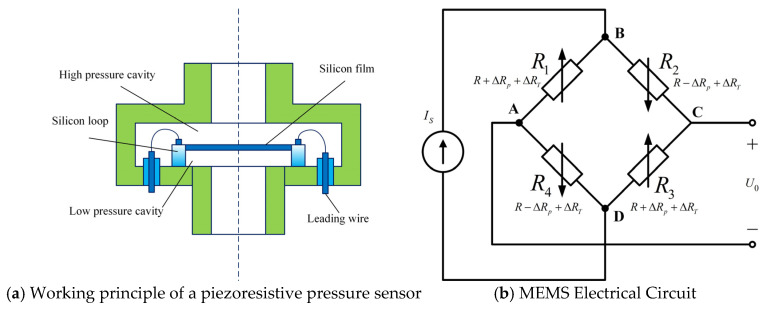
Working principle and measurement circuit of a piezoresistive pressure sensor.

Piezoresistive pressure sensors detect external pressure variations through the strain-resistance effect of the sensing material. As illustrated in Figure 1a, when external pressure is applied to the sensor diaphragm, the diaphragm undergoes slight deformation, causing strain-induced changes in the piezoresistive elements embedded on or within the diaphragm. Typically, four resistors are microfabricated on a silicon diaphragm and arranged along different directions of the diaphragm, forming a Wheatstone bridge that enables differential strain measurement and sensitivity optimization. Since the resistance of these elements is highly sensitive to deformation, it varies with changes in external pressure. To convert this mechanical deformation into an electrical signal, the piezoresistive elements are commonly connected in a Wheatstone bridge configuration, as shown in Figure 1b. When the external pressure changes, the bridge becomes unbalanced, generating a voltage output that exhibits a specific functional relationship with the applied pressure. This voltage signal is then amplified and conditioned by subsequent circuitry, making it available for the measurement system.

As illustrated in Figure 1b, the pressure sensor is typically configured as a full Wheatstone bridge, with terminals A and C serving as the output nodes, where the output voltage of the sensor is denoted as U_0_. Under balanced conditions, the resistances of all bridge arms are identical, as given in Equation (1).

(1) $$R=R_{1}=R_{2}=R_{3}=R_{4}$$

In the Wheatstone bridge circuit, the piezoresistive elements are symmetrically arranged, and under ideal conditions, each element has the same initial resistance *R*. The resistance variations induced by the applied pressure *P* have identical magnitudes, denoted as Δ*R_P_*, while the variations caused by temperature, denoted as Δ*R_T_*, are equal in both magnitude and sign for the two symmetric arms of the bridge. Therefore, when both pressure and temperature effects are applied simultaneously to the bridge, the resistance of each arm can be expressed as:

(2) $$\left\{\begin{array}{l} R_{1}=R+\Delta R_{p}+\Delta R_{T}\\ R_{2}=R-\Delta R_{p}+\Delta R_{T}\\ R_{3}=R+\Delta R_{p}+\Delta R_{T}\\ R_{4}=R-\Delta R_{p}+\Delta R_{T} \end{array}\right.$$

The output voltage of the pressure sensor is derived as presented in Equation (3):

(3) $$U_{0}=I_{s}\Delta R_{p}=I_{s}R(1+\alpha \Delta T)\pi \sigma$$

where $I_{s}$ denotes the constant current provided between terminals B and D; R represents the initial resistance of the strain resistors; α is the temperature coefficient of the piezoresistive elements; ΔT represents the ambient temperature variation; π denotes the silicon piezoresistive coefficient; and σ is the stress induced in the silicon diaphragm by the external pressure.

Moreover, the piezoresistive coefficient is affected by the ambient temperature.

(4) $$\pi =\pi_{0}(1+\beta \Delta T)$$

where $\pi_{0}$ denotes the piezoresistive coefficient at the reference temperature and β represents the thermal coefficient of piezo resistance.

Meanwhile, the mismatch in thermal expansion coefficients between the silicon substrate and the supporting beams leads to stress variations when the temperature changes [23]. The corresponding stress variation can be expressed as Equation (5):

(5) $$\Delta \sigma =\frac{\left(\alpha_{s}-\alpha_{g}\right)E_{0}\Delta T}{1+\mu }$$

where $E_{0}$ denotes the temperature coefficient of silicon (expressed in Kelvin), μ represents the Poisson’s ratio of silicon, and $\alpha_{s}$ and $\alpha_{g}$ correspond to the thermal expansion coefficients of silicon and the substrate, respectively. By combining Equations (3)–(5), the Wheatstone bridge output voltage is derived as shown in Equation (6):

(6) $$U_{0}=I_{s}R(1+\alpha \Delta T)\pi_{0}(1+\beta \Delta T)\left(\sigma +\frac{(\alpha_{s}-\alpha_{g})E_{0}\Delta T}{1+\mu }\right)$$

From Equation (6), it can be observed that the bridge output voltage is nonlinearly dependent on temperature variation, resulting in a pronounced temperature effect on the output signal. Hence, temperature compensation is essential to mitigate the temperature-induced deviation in pressure measurement and to enhance the accuracy and reliability of the sensor output.

### 2.2. Slime Mould Algorithm (SMA)

The Slime Mould Algorithm (SMA), inspired by the foraging behavior of slime moulds, is a representative swarm intelligence optimization algorithm. The algorithm exhibits emergent global behaviors at the population level through local interactions among individuals. The efficiency and effectiveness of swarm intelligence optimization largely depend on the balance between global exploration and local exploitation. Global search enables extensive exploration of the solution space to avoid local optima, while local search focuses on fine-tuning within a smaller region to accelerate convergence. Therefore, appropriately coordinating global exploration and local exploitation is key to enhancing overall algorithm performance.

During foraging, slime mould dynamically adjusts the parameters of its internal biological oscillators and the widths of its vein channels according to differences in food source concentration, forming an efficient food transport network. Notably, the channels connecting high-concentration food sources tend to be the widest. Based on an abstraction and simulation of this biological mechanism, researchers have developed a mathematical model of slime mould foraging behavior [24]. When approaching a food source, the position of the slime mould is updated according to the following Equation (7):

(7) $$X(t+1)=\left\{\begin{array}{l} X_{b}(t)+vb\cdot \left(W\cdot X_{A}\left(t\right)-X_{B}\left(t\right)\right),r<p\\ vc\cdot X(t),r\geq p \end{array}\right.$$

where p=tanh∣S(i)−DF∣ and $X_{b}(t)$ denotes the position corresponding to the optimal food concentration currently identified by the slime mould individual. vb is a randomly generated value oscillating within the range [−*a*, *a*]; W represents the food weight coefficient of the slime mould; $X_{A}\left(t\right)$ and $X_{B}\left(t\right)$ denote the positions of two individuals randomly selected from the population; vc is the search factor, a parameter that linearly decreases from 1 to 0 over the course of iterations, and r is a uniformly distributed random number within the interval [0, 1]. S(i) represents the fitness of the *i*-*th* slime mould individual, where i={1,2,…,N}. The update rule for a is defined as Equation (8):

(8) $$a=\mathrm{atanh}[-(t/max_{-}t)+1]$$

where $\max_{-}t$ denotes the maximum number of iterations and *t* represents the current iteration number. ***W*** simulates the variation in the propagation wave of the biological oscillator when the slime mould perceives different food concentrations, as expressed in Equation (9):

(9) $$W\left[sortS(i)\right]=\left\{\begin{array}{l} 1+r\times 1g\left(\frac{bF-S(i)}{bF-wF}+1\right),\;condition\\ 1-r\times 1g\left(\frac{bF-S(i)}{bF-wF}+1\right),\;others \end{array}\right.$$

where sortS(i) represents the sequence obtained by sorting the fitness values of all slime mould individuals, and *condition* denotes the first half of the sortS(i) sequence. *bF* and *wF* correspond to the best and worst fitness values during the convergence process, respectively.

During the foraging process, slime moulds also allocate a portion of individuals to perform random searches. Combining the aforementioned processes, the position update formula of the slime mould can be expressed as follows:

(10) $$X(t+1)=\left\{\begin{array}{l} rand\cdot (UB-LB)+LB,rand<z\\ X_{b}(t)+vb\cdot [W\cdot X_{A}(t)-X_{B}(t)],r<p\\ vc\cdot X(t),\;r\geq p \end{array}\right.$$

where *UB* and *LB* denote the upper and lower bounds of the search space for the slime mould individuals, respectively. *rand* is a random number uniformly distributed within the interval [0, 1], and z is a parameter representing the proportion of slime mould individuals performing random exploration, i.e., the mutation probability. This parameter ensures a good balance between global exploration and local exploitation. In this study, z is set to 0.03.

### 2.3. Modified Slime Mold Algorithm (MSMA)

To address the decline in the SMA’s search efficiency during later iterations—caused by its inherent mechanism—and its tendency to fall into local optima, as well as to improve the balance between its global exploration and local exploitation capabilities, this study proposes a Modified Slime Mould Algorithm (MSMA). The MSMA incorporates two key enhancement strategies:

1. Improving population initialization and diversity to enhance population quality. The initial population distribution strongly constrains the algorithm’s natural learning ability and its efficiency in locating the global optimum. Chaotic phenomena—characterized by highly unstable and unpredictable behavior within a deterministic system confined to a finite phase space—exhibit pseudo-randomness that makes them well suited for optimizing population initialization. Compared with traditional random initialization, chaotic initialization provides better internal structural properties, stronger ergodicity, more uniform distribution, and reduced repetitiveness in the search space. These advantages effectively enhance population diversity and quality, thereby improving the algorithm’s global exploration capability and accelerating convergence.

In this work, chaotic mapping is employed to optimize the initial distribution of the slime mould population. Among various chaotic maps, the Tent map produces populations with the most uniform and disordered distribution, ensuring both full search-space coverage and high uniformity. This contributes to stronger global exploration capability. The dynamic Tent chaotic distribution is expressed as in Equation (11):

(11) $$x_{n+1}=\left\{\begin{array}{l} x_{n}/0.7,x_{n}<0.7\\ 10\left(1-x_{n}\right)/3,x_{n}\geq 0.7,x_{0}\in [0,1],x_{n}\in (0,1) \end{array}\right.$$

where *n* denotes the current iteration step.

Tizhoosh [25] proposed the Opposition-Based Learning (OBL) method in 2005, which constructs an opposite solution corresponding to the current one during the search process and selects the better individual based on fitness comparison for the next iteration. This approach improves search efficiency and accelerates convergence toward the optimal solution. However, in multimodal optimization problems, the generated opposite solution may fall into other local optima, resulting in the loss of global information and potentially causing the algorithm to become trapped in a local optimum. To address this issue, the literature [26] introduced an elite individual mechanism into the traditional OBL framework and proposed the Elite Opposition-Based Learning (EOBL) strategy. This method retains elite individuals carrying key information during the iteration process and guides the population to approach these elites, thereby improving population quality and enhancing global optimization capability. Assume that the elite individual (i.e., the current optimal solution) in the slime mould population is denoted as $X_{i,j}\in \left\{X_{i,1},X_{i,2},\ldots X_{i,D}\right\}$, where *D* represents the dimensionality of the optimization problem. The elite opposite solution is defined by Equation (12):

(12) $$X_{i,j}^{*}=\alpha (LB_{j}+UB_{j})-X_{i,j},\;j=1,2,\ldots ,D$$

where $X_{ij}$ represents the value of the i-th dimension of the individual, α∈U(0,1), and $\left(LB_{j},UB_{j}\right)$ denote the dynamic lower and upper boundaries of the *j*-th dimensional search space, which are defined by Equation (13):

(13) $$\begin{array}{l} LB_{j}=\min(X_{i,j})\\ UB_{j}=\max(X_{i,j}) \end{array}$$

**Figure 2:**
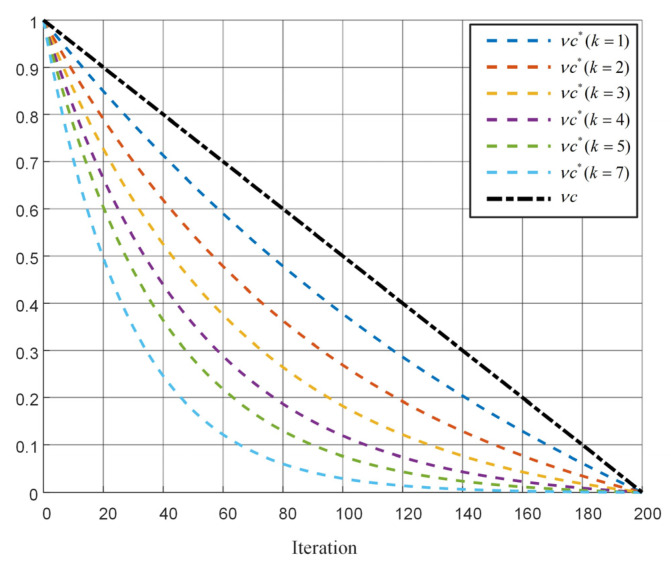
Variation of vc and $\nu c^{*}$ with iteration under different decay exponents *k*.

2. Adaptive adjustment of the search operator. In the standard SMA, the global search operator performs oscillatory exploration within a predefined range, while the local search operator follows a linearly decreasing strategy. The parameter νc, which governs the local exploitation capability in the later stages of iteration, decreases linearly with the iteration count. However, this linear attenuation is insufficient to simultaneously satisfy the requirement for large step sizes and rapid convergence during the early iterations, and small step sizes with slow, fine-grained exploration during the later iterations. To address this issue, an adaptive search factor is introduced. During the early stage of iteration, slime mold individuals must sense and explore the overall distribution of food concentrations in the global search space. Therefore, the search factor is designed to decrease rapidly, enabling large-step exploration. In contrast, during the later stage of iteration—when regions with high food concentration have been largely identified—the search factor decreases more gradually, facilitating fine-grained exploitation around promising regions. Furthermore, an exponential term *k* is incorporated into the search factor to flexibly adjust the attenuation rate, allowing the algorithm to dynamically regulate the local search step size based on the current position within the iteration process. The adaptive search factor $\nu c^{*}$ is defined as shown in Equation (14).

(14) $$\nu c^{*}={\left[(e^{(t_{\max}-t)/t_{\max}}-1)\cdot \frac{1}{e-1}\right]}^{k}$$

where *t* denotes the current iteration number, $t_{\max}$ represents the maximum number of iterations, The parameter *k* represents the exponent controlling the decay rate. The variation trends of the original search factor and the proposed adaptive search factor under different values of *k* are illustrated in Figure 2. As illustrated, excessively large or excessively small values of *k* fail to satisfy the dual requirements of the algorithm—namely, large step sizes during the early stage of iteration and small step sizes during the later stage. Such imbalance may reduce the convergence speed or cause the algorithm to become trapped in local optima, thereby hindering performance improvement. Through extensive comparative experiments conducted with different values of *k*. It was observed that* k* = 4 provides the most balanced variation in the adaptive search factor, effectively enhancing the trade-off between global exploration and local exploitation. Therefore, a decay-rate exponent of 4 is adopted in this study.

Substituting it into the third equation of Equation (10), the position update formula can be obtained as shown in Equation (15).

(15) $$X(t+1)=\nu c^{*}\cdot X(t),\;r\geq p$$

### 2.4. Performance Evaluation on Benchmark Functions

To evaluate the performance of MASM, simulation verification was conducted. The simulation testing software and version used were MATLAB 2024b, and the computer configuration was ULTRA with a 7-core 155H CPU, 32 GB of RAM, and an NVIDIA RTX 500 Ada GPU. During the test verification, each algorithm was run independently 50 times, and the test results of each algorithm were recorded and averaged. The opulation size of the algorithm and the maximum number of iterations were preset as 30 and 300 respectively.

To verify the global search ability, local search ability, ability to escape from local optimum, and optimization accuracy of the proposed algorithm, this paper selected ten common standard test functions (Sphere, Schwefel2.22, Easom, Schwefel2.21, Quartic, Bridge, Generalized Rosenbrock, seven functions) to test the local development ability of the algorithm, and three functions (Schwefel2.26, Generalized, Levy 1.3, Ackley) to test the ability of the algorithm to escape from local optimum and the switching ability between global exploration and local search), with the minimum value of the benchmark test function as the target value of the fitness function. The details of the functions are shown in Table 2. Simulation testing verification was carried out using MSMA, and the optimization results of MSMA and SMA were compared with those of 5 types of algorithms (SA, AOA, GA, GWO, and PSO).

**Figure 3:**
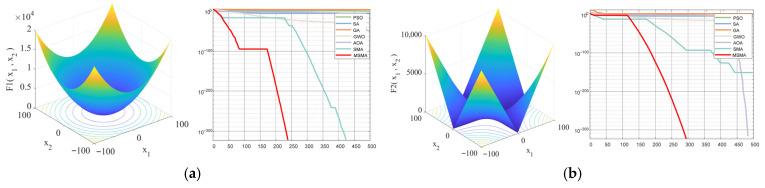

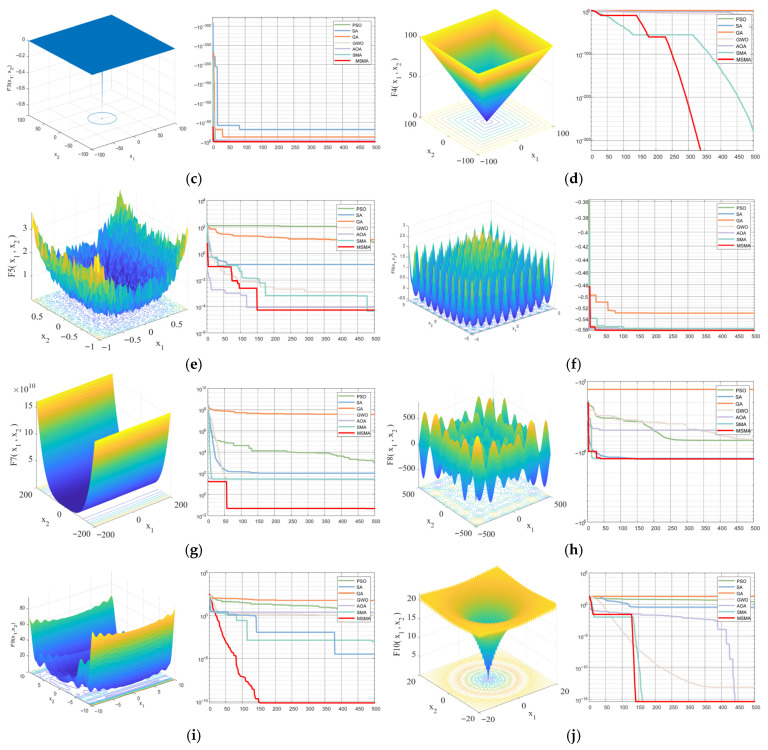
The convergence curve of fitness value of optimization process under different test functions. (**a**−**j**) respectively represent the test functions F1−F10.

From Figure 3 , it can be observed that MSMA performs well on unimodal functions (F1–F4), exhibiting high convergence accuracy, strong stability, and fast convergence speed, while also demonstrating the capability to escape local optima. For multimodal functions (F5–F7), MSMA shows a relatively slower convergence rate; however, its final convergence accuracy remains superior to that of the other algorithms. When dealing with complex, highly irregular multimodal functions with substantial interference (F9–F10), MSMA demonstrates strong adaptability, effectively escaping local optima to a certain extent while maintaining the stability of the obtained optimal solutions.

Compared with the original SMA, MSMA achieves improvements in both convergence speed and solution accuracy. In comparison with GA and PSO, the proposed MSMA converges faster, requires fewer control parameters, and exhibits stronger stability. In particular, GA is prone to premature convergence and low optimization accuracy, making it less effective for multimodal optimization tasks. These results collectively indicate that the introduced optimization strategies significantly enhance the computational efficiency and global search capability of SMA, enabling MSMA to exhibit greater robustness and higher solution efficiency when handling complex optimization problems.

**Figure 4:**
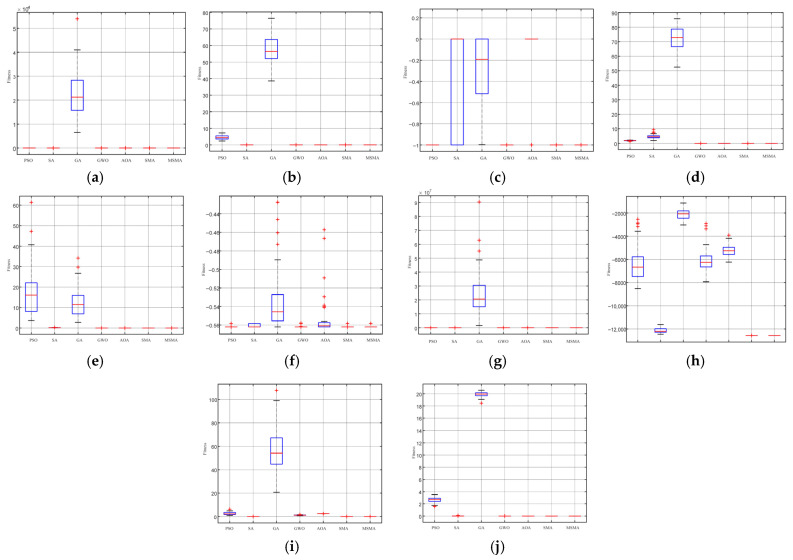
Boxplots of optimization results for different algorithms at dim = 30 over 50 independent runs (500 iterations). (**a**−**j**) respectively represent the test functions F1−F10.

Figure 4 highlights clear differences in algorithm performance distributions within the 30-dimensional search space. MSMA exhibits a smaller box height, indicating lower dispersion and thus stronger stability and robustness. Its median value is consistently lower than those of competing algorithms, reflecting superior overall optimization performance. Additionally, shorter whiskers and fewer outliers suggest more consistent results across independent runs and reduced sensitivity to randomness. In contrast, several comparison algorithms show larger interquartile ranges and more outliers, implying higher variability and poorer stability, likely due to premature convergence or entrapment in local optima—a challenge that becomes more pronounced in high-dimensional complex problems.

### 2.5. Ablation Study on Benchmark Functions

To analyze the independent contributions of each improvement strategy incorporated in the proposed MSMA, an ablation study is conducted. Based on the original Slime Mould Algorithm (SMA), MSMA introduces several enhancement mechanisms, including chaotic initialization, an adaptive search operator (learning factor), and elite opposition-based learning. Multiple algorithmic variants are constructed, consisting of the original SMA and seven different combinations of the proposed improvements.

For clarity in presenting the ablation results, unified code identifiers are assigned to each algorithm variant as follows: M0: Original SMA (baseline); M1: Full MSMA (M2 + M3 + M4); M2: SMA + Chaotic Initialization; M3: SMA + Adaptive Search Operator; M4: SMA + Elite Opposition-based Learning; M5: SMA + Chaotic Initialization + Adaptive Search Operator (M2 + M3); M6: SMA + Chaotic Initialization + Elite Opposition-based Learning (M2 + M4); M7: SMA + Adaptive Search Operator + Elite Opposition-based Learning (M3 + M4). Each algorithm is independently executed 30 times on every benchmark function. The population size is set to 30, and the maximum number of iterations is fixed at 500. The best fitness value obtained in each run is recorded, and the mean and standard deviation are finally computed as the performance evaluation metrics.

As shown in Table 3, different improvement modules have a significant impact on the optimization performance of SMA. For some relatively simple functions (F2–F4), most algorithms are able to reach or approach the theoretical optimum; however, noticeable differences in stability exist among the variants. Compared with the baseline algorithm M0, algorithms incorporating improvement strategies generally exhibit smaller standard deviations, indicating more stable convergence results.

For multimodal functions F5–F7, M0 shows relatively higher mean values and larger standard deviations, suggesting that it is prone to being trapped in local optima and exhibits strong stochastic fluctuations. Variants with a single improvement strategy (M2–M4) display inconsistent performance across different functions, indicating that an individual mechanism alone cannot comprehensively enhance algorithm performance and may even degrade the accuracy of M0 in some cases. In contrast, multi-strategy variants (M5–M7) achieve lower mean values and smaller standard deviations on most functions, demonstrating stronger global search capability and stability.

For complex multimodal functions F8–F10, the performance differences become even more pronounced. M0 performs poorly on F8, whereas algorithms incorporating improvement mechanisms—particularly those including chaotic initialization—significantly reduce the objective function values, indicating that this strategy effectively enhances global exploration ability. On F9 and F10, most improved algorithms converge stably to solutions close to the optimum, while the baseline algorithm still exhibits considerable variability.

The proposed MSMA achieves the best or near-best results on the majority of benchmark functions with relatively small standard deviations, demonstrating strong synergy among the improvement strategies. The ablation results clearly confirm that the introduced mechanisms—chaotic initialization, the adaptive search operator, and elite opposition-based learning—each contribute positively to improving SMA performance, while their integration further enhances global optimization capability and stability. Overall, the experimental results verify the effectiveness of each module and show that their combined application significantly strengthens the optimization ability of the original SMA, thereby demonstrating the rationality and effectiveness of the proposed MSMA design.

### 2.6. Temperature Compensation Algorithm Based on MSMA-BP

The essence of the temperature compensation algorithm for pressure sensors lies in correcting the nonlinear output errors induced by temperature variations. Feedforward neural networks are capable of performing arbitrary nonlinear mappings between inputs and outputs, making them highly suitable for nonlinear fitting tasks. Among them, the BackPropagation (BP) neural network can effectively model complex nonlinear relationships while featuring a simple architecture and ease of engineering implementation.

**Figure 5:**
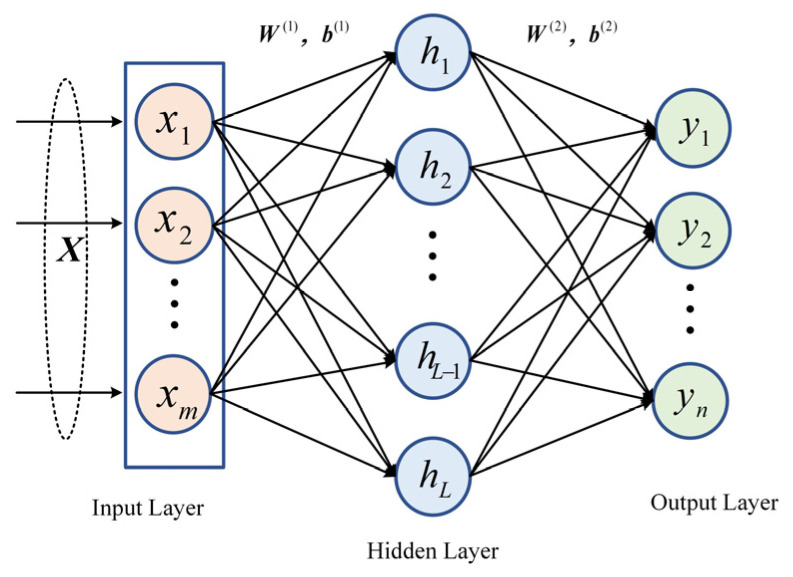
BP Structure Diagram.

The BP neural network is a typical multilayer feed-forward architecture, and its structural schematic is illustrated in Figure 5. The network consists of an input layer, a hidden layer, and an output layer. Signals are propagated between layers through weighted connections, while nonlinear mapping is achieved by activation functions. The output of the network corresponds to the compensated pressure. Assuming that the hidden layer contains *L* neurons and the training dataset consists of *S* samples, $\left(X,T\right)=\{\left(\begin{array}{c} x_{i},t_{i} \end{array}\right)|x_{i}\in {\mathbb{R}}^{m},t_{i}\in {\mathbb{R}}^{n},i=1,2,\ldots ,S\}$. Here, *m* and *n* denote the number of neurons in the input layer (i.e., the feature dimension of the samples) and the number of neurons in the output layer (i.e., the dimension of the sample labels), $x_{i}={\left(\begin{array}{c} x_{i1},x_{i2},\ldots ,x_{im} \end{array}\right)}^{T}$ represents the feature vector of the *i*-th sample with length *m*, $t_{i}={\left(\begin{array}{c} t_{i1},t_{i2},\ldots ,t_{in} \end{array}\right)}^{T}$ denotes the corresponding label of the *i*-th sample, and $y_{i}={\left(\begin{array}{c} y_{i1},y_{i2},\ldots ,y_{in} \end{array}\right)}^{T}$ denotes the output of the network for the *i*-th sample. The relationship between the input and output of the BP neural network is given by Equation (16).

(16) $$y_{i}=W^{\left(2\right)}g\left(W^{\left(1\right)}x_{i}+b^{\left(1\right)}\right)+b^{\left(2\right)},i=1,2,\ldots ,S$$

where $W^{\left(1\right)}$ denotes the weights of the input layer and hidden layer, $b^{\left(1\right)}$ represents the bias of the hidden layer, $W^{\left(2\right)}$ denotes the weights connecting the hidden layer to the output layer, $b^{\left(2\right)}$ is the bias of the output layer, and g represents the activation function.

However, during the training process of the BP neural network, issues such as poor stability and local optima easily occur. The main reason lies in the strong dependence of network training on the initial weights and thresholds, which are typically set randomly, leading to uncertainty in the convergence direction. To address these issues, the MSMA is employed to perform global optimization of the initial weights and thresholds, thereby enhancing the convergence capability of the model. In this study, an improved SMA, termed MSMA, is proposed to optimize the initial parameters of the BP neural network. The optimized BP network is then utilized for temperature compensation of the pressure sensor, effectively improving its measurement accuracy. The specific procedure of the pressure sensor temperature compensation model based on MSMA-BP is illustrated as follows.

Step 1: Set the maximum number of iterations $t_{\max}$, population size n, and the upper and lower bounds of the solution space UB and LB. Use Tent chaotic mapping to initialize the population distribution to ensure a more uniform coverage of the search space;

Step 2: Enter the iteration loop. Compute the fitness of each individual, identify the current best fitness bF and worst fitness wF of the population, and substitute them into Equation (9) to calculate the weight coefficient W. Record the current best position $X_{b}$ and the optimal fitness DF. Then, calculate parameter a using Equation (8) and generate a random number rand;

Step 3: If rand < 0.03 and i < n, he slime mold population will perform a random search behavior according to Equation (4), expanding the search range until the condition rand ≥ 0.03 is met;

Step 4: If rand ≥ 0.03, update the parameters p, vb and the adaptive factor $\nu c^{*}$;

Step 5: If r < p, the slime mold population executes a global search based on Equation (10), narrowing the search scope and moving toward areas with higher food concentration;

Step 6: If r ≥ p, the slime mold population performs local search using Equation (15) with the adaptive factor $\nu c^{*}$, adjusting step size adaptively to explore smaller regions with high food concentration for better optima.

Step 7: Generate an elite opposition-based solution according to Equation (12), compare it with the current best solution, and retain the superior individual for the next iteration.

Step 8: Check whether the maximum iteration condition is met. If the current iteration number t reaches $t_{\max}$, stop the iteration and output the optimal position $X_{b}$, best fitness DF, and optimal food concentration. Otherwise, return to Step 3 and continue the loop.

Step 9: Output the position corresponding to the global optimal fitness and use it to initialize the BP network’s initial weights and thresholds.

**Figure 6:**
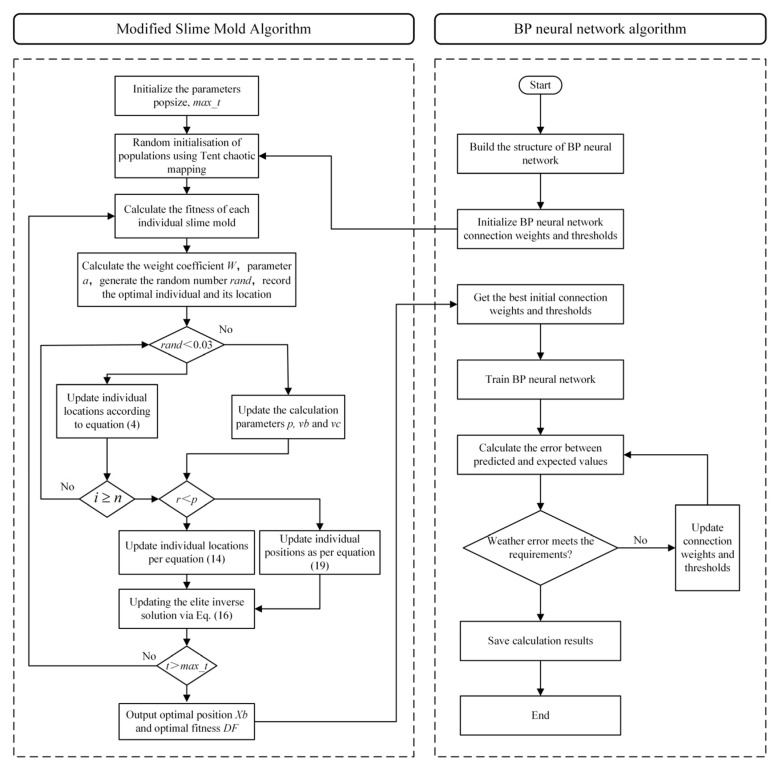
Flowchart of MSMA-BP.

Step 10: Train the BP neural network using the weights and thresholds obtained in Step 9 until the test error meets the accuracy requirements. The resulting network constitutes the MSMA-BP temperature compensation model. The flowchart of the MSMA-BP-based temperature compensation algorithm is shown in Figure 6.

## 3. High-Temperature Experiments and Data Characteristic Analysis

To address the significant temperature drift exhibited by sensors under high-temperature and high-pressure conditions in ultra-deep drilling scenarios, this study conducted combined temperature–pressure calibration experiments on the sensors in a laboratory setting to obtain training data for the compensation model. To ensure that the experimental data reflect the actual field environment, this section provides a detailed description of the experimental conditions and procedures.

### 3.1. Calibration Experiments of the Pressure Sensor

In real ultra-deep drilling environments, pressure sensors are typically subjected to multiple challenges, including high temperatures, high pressures, and intense vibrations. Downhole pressure increases with depth and can reach up to 170 MPa, while the bottom-hole temperature can reach 175 °C and may vary slowly due to the circulation of drilling fluid and heat exchange with the formation. Additionally, complex operational conditions during drilling—such as drill-string vibrations, bottom-hole pressure pulsations, and impacts from fluid flow—can significantly affect sensor output, resulting in non-ideal characteristics such as temperature drift, hysteresis errors, and signal fluctuations over long-term operation. Compared with actual downhole conditions, the laboratory environment is more stable and controllable. By performing complete pressure-loading experiments at multiple temperature points, the main error trends of the sensor under high-temperature and high-pressure conditions can be systematically captured, providing reliable foundational data for constructing a temperature compensation model. Furthermore, when combined with subsequent embedded testing, these experiments allow further validation of the compensation method under conditions closer to actual field applications.

**Figure 7:**
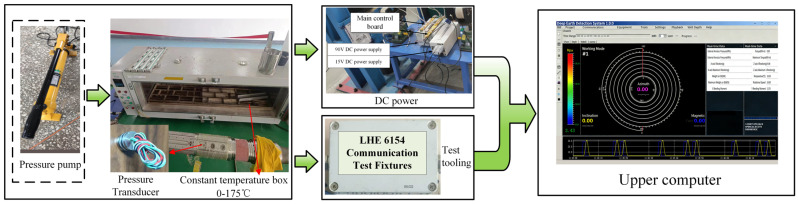
Experimental setup used for temperature-compensation testing of the MEMS pressure sensor.

**Figure 8:**
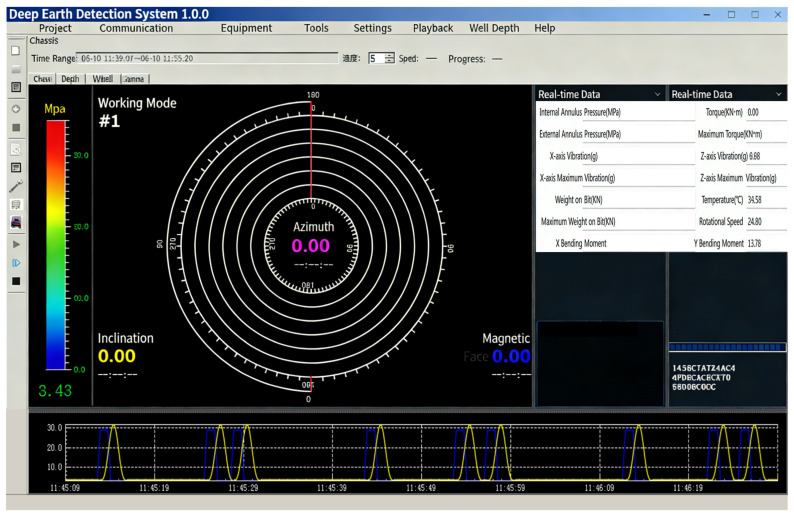
Interface of the DEES software.

The experimental setup consists of a pressure-loading system, a pressure sensor, a data acquisition and communication module, a high-temperature environment simulation system, and a PC-based software platform. The main components of the test bench are summarized in Table 4, and the overall framework is illustrated in Figure 7. The pressure loading is provided by a constant-pressure pump fixture, capable of delivering stable and controllable pressure output in the range of 0–170 MPa. The tested MEMS pressure sensor is connected via wires to the LHE6154.221a master/control and communication test module, which uses the RS485 protocol to acquire sensor signals, perform data packaging, and transmit data in real time. After being mounted on the test fixture, the sensor is placed inside a high-temperature test chamber to simulate downhole high-temperature conditions. The PC hosts the Deep Earth Exploration System (DEES) software (v1.0.0), which is responsible for real-time monitoring of pressure and temperature signals, configuration of experimental parameters, and data recording. The software interface and settings are shown in Figure 8. All test equipment is integrated at the system level through unified power supply and communication links, thereby establishing a complete experimental platform for conducting pressure–temperature coupled tests.

A high-temperature chamber combined with a pressure pump was employed to conduct the temperature–pressure coupled loading test on the pressure sensor. Prior to the experiment, the pressure pump fixture was connected to the pressure sensor and mounted inside the test sub. The system pressure was then released to zero to ensure an accurate and reliable initial state. Afterward, the entire pressure-loading assembly was placed inside the high-temperature chamber, and the heating rate was set to 6 °C/10 min. Once the chamber reached the target temperature, a 1 h dwell was applied to ensure thermal equilibrium between the sensor interior and the ambient environment. When the temperature stabilized at 25 °C, the first pressure calibration was performed. Pressure was gradually increased using the pressure pump in steps of 0 MPa, 20 MPa, 40 MPa, 60 MPa, 80 MPa, 100 MPa, 120 MPa, 140 MPa, 160 MPa, and 170 MPa. At each pressure level, the sensor output was recorded after the measurement stabilized. Data acquisition was completed using the LHE6154 acquisition fixture and the DEES.

**Figure 9:**
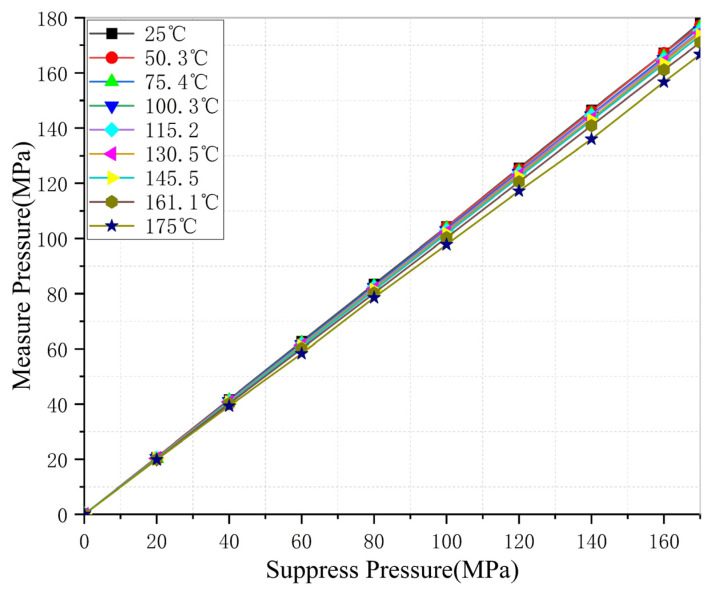
Non-compensated output characteristics of the MEMS piezoresistive pressure sensor.

The procedure was subsequently repeated at setpoint temperatures of 50, 75, 100, 115, 130, 145, 160, and 175 °C. It should be noted that the temperatures reported in Figure 9 correspond to the actual stabilized temperatures measured at each setpoint, which were 25, 50.3, 75.4, 100.3, 115.2, 130.5, 145.5, 161.1, and 175 °C, respectively. The slight deviations from the nominal setpoints are attributable to the chamber’s temperature control limitations and fall within acceptable experimental tolerances. In total, 90 data points were collected across nine temperature levels and ten pressure levels, providing a comprehensive dataset for training the compensation model.

As shown in Figure 9, prior to compensation, the absolute value of the pressure measurement error gradually decreases with increasing temperature, ranging from 3.3 to 8.3 MPa, with a maximum full-scale error of 4.88%. When the temperature reaches 175 °C, the maximum error reduces to 3.3 MPa. The temperature coefficient of sensitivity (TCS) reaches a maximum of 0.812%FS/°C. As the temperature increases, the TCS decreases at higher temperatures.

**Figure 10:**
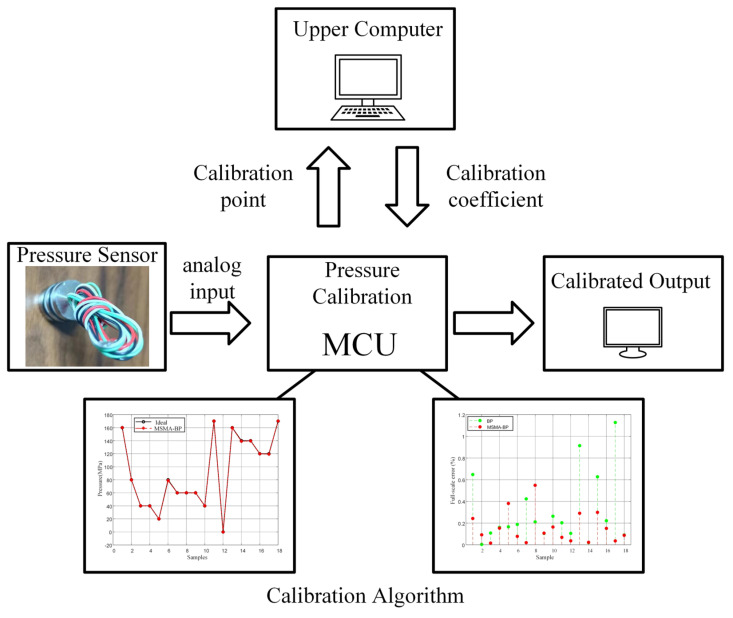
The workflow of the software-based compensation.

The workflow of the software-based compensation is illustrated in Figure 10. An effective algorithm is critical for improving sensor output accuracy. Under a preset pressure gradient, the sensor output signals at different temperatures are acquired. The analog pressure and temperature signals are converted to digital values via an analog-to-digital converter and stored in the host computer. The host computer then uses the experimentally collected pressure and temperature data to train the neural network model, computing the optimal correction parameters (weights and biases). The trained model parameters are subsequently exported to a storage unit for use by the microcontroller (MCU). During system operation, the MCU retrieves the stored neural network parameters to perform real-time compensation on the sensor output, generating calibrated pressure data. In this way, the pressure sensor can achieve offline intelligent calibration.

### 3.2. Analysis of Correction Results

90 experimental samples were processed, and the proposed temperature compensation method was validated. The data were first standardized using the Z-score method to eliminate the influence of measurement units and improve model training stability. Subsequently, the dataset was randomly split into training and test sets at an 8:2 ratio. A BP neural network model was established, and the Modified Slime Mold Algorithm (MSMA) was employed to optimize the initial weights and biases of the BP network. The standardized training set was then used to train the model, further enhancing the network’s convergence performance and the accuracy of sensor pressure calibration. The BP network architecture and the MSMA initialization parameters are listed in Table 5.

**Figure 11:**
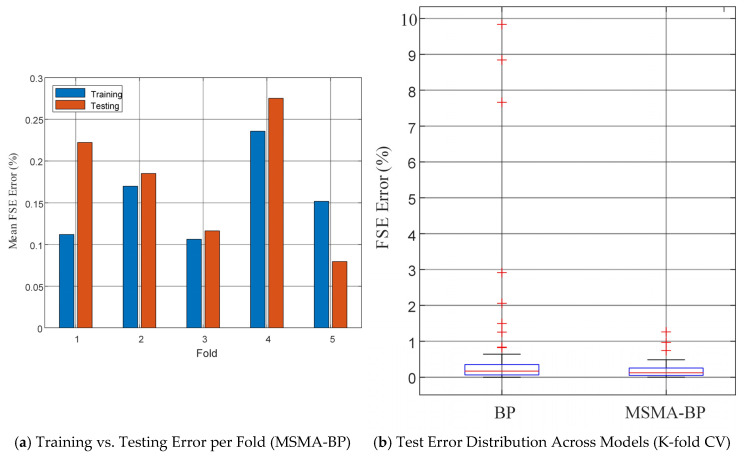
The training error and test error of MSMA-BP at each fold.

Figure 11a shows a bar chart comparing the training and testing errors of MSMA-BP across different folds. It can be seen that the difference between training and testing errors is extremely small across all folds (with a maximum error of 0.11% on both the training and validation subsets), and in the 5th fold, the testing error is even lower than the training error. This indicates that the model’s performance on unseen data is essentially consistent with that on the training data, and there is no instance where the training error is significantly lower than the testing error. Therefore, it can be concluded that the designed neural network does not exhibit overfitting and possesses good generalization ability. Figure 11b shows the box-and-whisker plots of the K-fold test error distributions for the models (original BP, MSMA-BP). The results indicate that the test error of the MSMA-optimized model is significantly reduced, and the range of error fluctuations is also smaller, demonstrating that the improved method can stably enhance the calibration accuracy of pressure sensors under small-sample conditions. These results validate the stability and reliability of the proposed method across the full measurement range and further demonstrate the model’s predictive capability for various temperature–pressure combinations.

**Figure 12:**
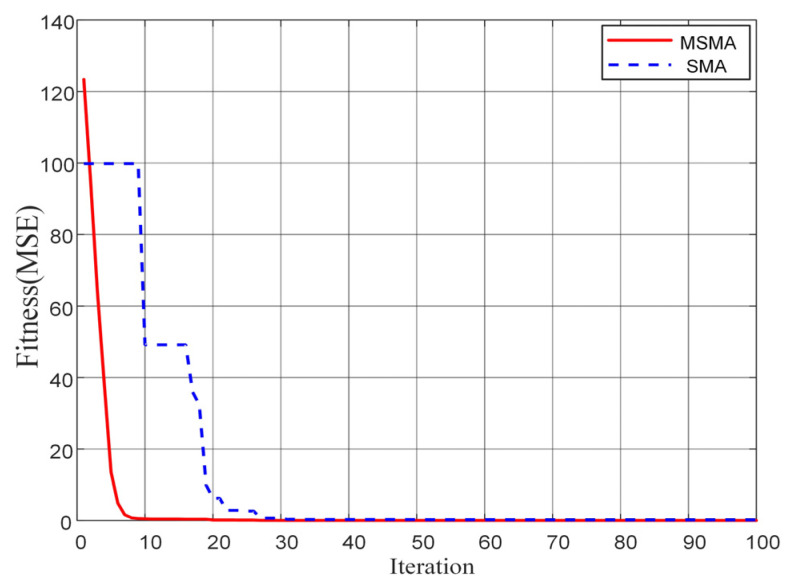
Schemes follow the same formatting.

To evaluate the effectiveness of MSMA in the optimization process, it was compared with the original Slime Mold Algorithm (SMA) in terms of convergence speed, global search capability, and the ability to avoid local optima. This comparison provides a clear demonstration of the improvements introduced by the modified strategy in both convergence efficiency and search performance. Figure 12 illustrates the iterative convergence process of the algorithms. During the population iterations, the maximum mean squared error (MSE) was used as the fitness function. As shown in the figure, the MSMA population reaches the optimal fitness value after 10 iterations, whereas the SMA requires 30 iterations to achieve the optimum, with multiple instances of stagnation in local optima during convergence. The results indicate that MSMA exhibits superior performance over SMA in terms of faster convergence and enhanced ability to escape local optima.

**Figure 13:**
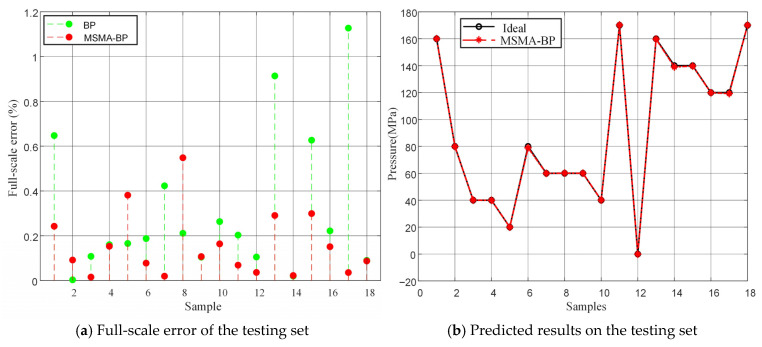
Full-scale error and predicted results on the test set.

As shown in Figure 13 and Table 6, there are significant differences between the full-scale error characteristics and pressure prediction results of BP and MSMA-BP. The BP method exhibits a maximum error of 1.15% and an average error of 0.30%, with high error variability, indicating limited compensation stability under complex temperature conditions. In contrast, the MSMA-BP method exhibits a maximum error of 0.51% and an average error reduced to 0.15%, with a distribution showing lower bias and higher concentration overall. This demonstrates the effectiveness of MSMA in suppressing local optima and learning sensor data characteristics. This indicates that MSMA-BP possesses higher compensation accuracy and robustness when handling temperature-induced strong drift, and can significantly improve the compensation accuracy of MEMS piezoresistive pressure sensors in high-temperature environments in ultra-deep wells.

To comprehensively evaluate the performance of the proposed MSMA method for temperature compensation of pressure sensors under ultra-deep well high-temperature and high-pressure conditions, several typical methods—including polynomial fitting, linear interpolation n, ELM, RELM, LSSVM, and BP—were selected for comparison. Among all comparison methods, the model architecture and training hyperparameters remain consistent.

To eliminate random effects, all algorithms were independently executed 30 times on the same dataset. The mean ± standard deviation (mean ± std) and the best single-run results of the full-scale maximum error (Max FS Error) and full-scale average error (Avg FS Error) were calculated, and the average computation time during both training and testing phases was recorded. Detailed results are presented in Table 7.

As shown in Table 7, under ultra-deep well high-temperature and high-pressure conditions, traditional interpolation and polynomial methods perform poorly, exhibiting generally high errors with considerable fluctuations. Specifically, linear interpolation yields a maximum error of 5.3% ± 3.8%, indicating relatively low overall accuracy, while polynomial interpolation shows slight improvement with a maximum error of 1.27% ± 0.70%, yet it still fails to meet high-precision compensation requirements. In comparison, methods such as Gaussian Process Regression (GA) ELM, RELM, LSSVM, and BP achieve significantly lower average errors than interpolation-based approaches; however, their stability varies, and prediction results show notable fluctuations. The BP network produces a maximum error of 1.15% ± 1.04% (best: 0.36%) and an average error of 0.30% ± 0.16%, but the relatively high standard deviation (1.04%) indicates insufficient stability and considerable result variability. The proposed MSMA-BP model demonstrates optimal performance in both accuracy and stability. Across 30 independent runs, the model achieves a maximum error of 0.74% with a standard deviation of 0.23%, and an average error of 0.26% with a standard deviation of 0.03%, showing the smallest variability and the most concentrated distribution of results. The integration of MSMA significantly improves the convergence characteristics of the BP network, enabling high-precision and stable compensation under high-temperature nonlinear drift, thereby demonstrating strong robustness and practical applicability.

Regarding computational efficiency, the training time of MSMA-BP is 2.53 s, which is higher than that of conventional methods. This is primarily due to the multiple population-based iterations in MSMA for optimizing network weights and biases to obtain the global optimum. Although this increases computational demand, it effectively mitigates instability from random initialization and allows sufficient exploration of the search space, enhancing both prediction accuracy and generalization capability. In contrast, the RELM method bypasses repeated iterative training over the dataset by computing the weights in a single step through the generalized inverse, offering higher computational efficiency in structure. While the incorporation of MSMA increases training time, the majority of computational load occurs during the optimization process rather than repeated network training. During testing, the average inference time of MSMA-BP is 85.5 ms, comparable to ELM and LSSVM, and is sufficient to meet real-time requirements in engineering applications. Although traditional interpolation methods achieve the fastest testing speed (0.02 ms), their low accuracy limits applicability under complex conditions. Overall, the MSMA-BP method achieves an effective balance among accuracy, stability, and real-time performance under ultra-deep well high-temperature and high-pressure environments.

**Figure 14:**
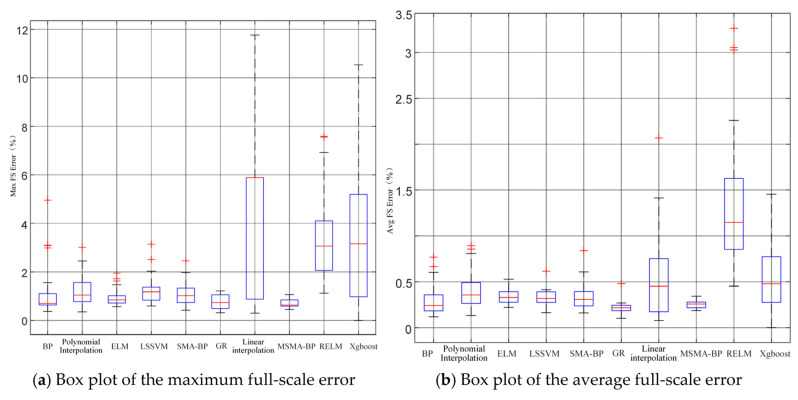
Box plots of compensation errors for different methods.

The mean and standard deviation can provide an overall description of the compensation results, but they are insufficient for effectively detecting outliers. To offer a more intuitive representation of the central tendency and dispersion of the compensation results, box plots were drawn, as shown in Figure 14. In practical applications, model training is typically performed offline on a PC or server, while testing and prediction tasks are executed on-site. Since the inference stage of the HLEWOA-RELM model only involves matrix operations, its testing time (117 ms) is comparable to that of ELM and LSSVM, which is sufficient to meet the requirements of downhole compensation. Therefore, although the training stage is slightly longer, the computational demand is feasible under current hardware conditions while providing improved convergence performance and more stable predictions. The proposed HLEWOA-RELM model can be implemented in an “offline training—online application” engineering scenario, achieving a balance between high accuracy and real-time performance and demonstrating good potential for practical deployment and scalability.

**Figure 15:**
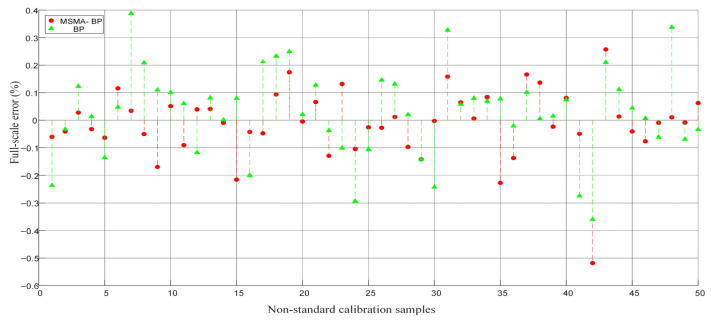
Data fitted to non-calibration points.

Generate 50 non-calibrated data points within the calibrated temperature–pressure range, use the model to make predictions, and compare the results with the theoretical output obtained via linear interpolation to calculate the full-scale error at each point. All out-of-range data points are located within the convex hull of the original experimental data, ensuring that the predicted points fall within the coverage of the existing samples and avoiding the uncertainty associated with extrapolation. As shown in Figure 15, the maximum error of the MSMA-BP model at out-of-range data points is 0.38%, with an average error of 0.10% and a standard deviation of 0.14%; while the BP model exhibits a maximum error of 0.54%, an average error of 0.15%, and a standard deviation of 0.14%. The results indicate that the MSMA-BP model maintains high-precision predictions at non-calibration points while effectively suppressing error fluctuations, demonstrating greater stability compared to the original model. Overall, this method exhibits good interpolation capability and generalization performance under continuous temperature–pressure conditions. The partial correction situation is shown in Table 8.

The error at non-calibrated points and the error amplitude at calibrated points remain at the same level, indicating that the model has good interpolation capability within the sample convex hull and does not exhibit significant error growth when moving away from the training grid. Overall, MSMA-BP maintains high-precision predictions at non-calibrated points while effectively suppressing error fluctuations, demonstrating greater stability compared to the original model. The results further validate the interpolation accuracy and generalization capability of the proposed method under continuous temperature–pressure conditions.

To evaluate the robustness of the proposed method under different noise conditions, this study constructed a noisy training dataset by superimposing Gaussian white noise with varying signal-to-noise ratios (SNRs = −10, −5, 0, 5, and 10 dB) onto the outputs of the original pressure sensor training set, thereby simulating the interference and errors encountered in underground pressure measurements. All data were processed identically at each noise level. Each model was trained on the noisy training set and tested on the test set. To ensure the reproducibility of results, independent and fixed random seeds were used for random initialization at each noise level. Model prediction accuracy was evaluated using the mean squared error (MSE) and full-scale error percentage (FS Error, %).

**Figure 16:**
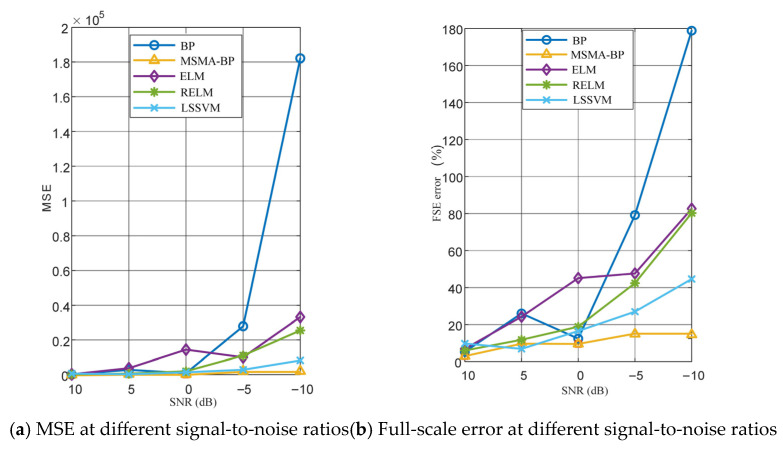
Comparison of the Performance of Various Prediction Models at Different Signal-to-Noise Ratios.

As shown in Figure 16, there are significant differences in the sensitivity to noise among the various models. At higher signal-to-noise ratios (10 dB and 5 dB), all models maintain low prediction errors and demonstrate good fitting capabilities. Under high noise levels (SNR = −10 dB), the MSMA-BP model exhibits excellent noise robustness. The above results indicate that MSMA-BP and LSSVM exhibit stronger noise resistance than the comparison methods across all tested noise levels. This may be attributed to the risk minimization principle in the LSSVM architecture and the global optimization of initial weights in the BP network by the MSMA, enabling the models to effectively capture the intrinsic mapping relationship between inputs and outputs in noisy training data. Full-scale error: As the signal-to-noise ratio decreases, the full-scale error tends to increase. Under strong noise conditions, the MSMA-BP method exhibits the lowest full-scale error compared to the other methods.

### 3.3. Embedded Pressure Sensor Temperature Drift Compensation Verification

Based on the software compensation scheme described above, performance testing experiments were conducted for the calibration algorithm integrated into the embedded system. The setup for the experimental apparatus was consistent with that used in the calibration tests. The sensors used during the validation phase were from the same production batch and model as those used in the calibration experiments, ensuring consistency in their characteristics.

The temperature compensation model is deployed on the STM32F407 microcontroller (ARM Cortex-M4, 168 MHz, STMicroelectronics, Geneva, Switzerland). It occupies approximately 0.16 KB of Flash memory, which is less than the MCU’s available resources (1 MB Flash, 192 KB SRAM), making it suitable for resource-constrained downhole environments. The single-sample inference latency measured on the MCU averages 3.17 ms (excluding communication time), meeting the real-time requirements for high-frequency drilling data processing.

During testing, the temperature chamber was sequentially set to 25 °C, 60 °C, 100 °C, 130 °C, and 175 °C. At each temperature setting, the applied pressure was adjusted to 0, 20, 60, 100, 140, 160, and 170 MPa, and the corresponding sensor outputs were recorded. Table 9 presents the measured pressure values of the sensor under different temperature conditions.

For temperatures below 100 °C, the maximum applied pressure was set to 140 MPa. This range exceeds the typical formation pressure encountered in deep drilling operations (generally below 120 MPa), thereby ensuring a comprehensive evaluation of the sensor’s measurement performance under representative formation pressure conditions.

**Figure 17:**
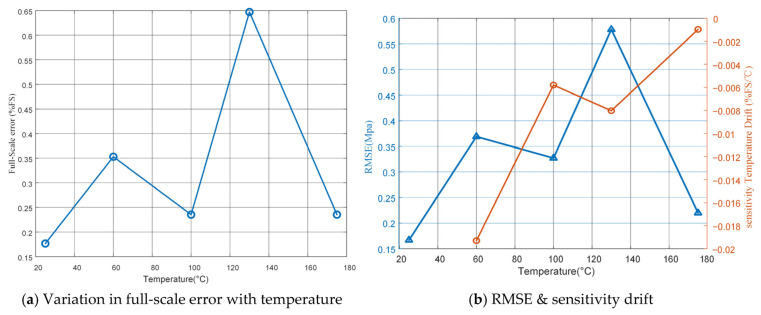
Comprehensive comparison of compensation performance indicators.

As shown in Table 9 and Figure 17a, the measurement errors at most temperature points remain below 0.4% FS. At 130 °C and 140 MPa, the pressure sensor exhibits the largest deviation, with a maximum error of 1.1 MPa, corresponding to a full-scale maximum error of 0.64%.

To further evaluate the measurement accuracy after temperature compensation, Figure 17b presents the root mean square error (RMSE) and the sensitivity temperature drift at different temperatures. The RMSE reflects the overall deviation between the sensor output and the true pressure; a smaller RMSE indicates higher measurement accuracy of the compensated sensor output. The sensitivity temperature drift represents the influence of temperature on the sensor’s sensitivity, with 25 °C used as the reference temperature. The calculation methods are given in Equations (17) and (18).

(17) $$\mathrm{RMSE}=\sqrt{\frac{1}{N}\sum_{i=1}^{N}{\left(P_{m}-P_{i}^{*}\right)}^{2}}$$

(18) $$\alpha_{s}(T)=\frac{S_{T1}-S_{T}}{S_{T}\cdot (\Delta T)}\times 100\%$$

where N is the number of measurement points, $P_{m}$ is the compensated pressure output, $P_{i}^{*}$ is the corresponding true pressure, $S_{T1}$ denotes the sensitivity at temperature T1, $S_{T}$ is the sensitivity at the reference temperature, and ΔT represents the temperature variation.

After compensation, the RMSE values are all below 0.6 MPa, with the minimum value of 0.22 MPa observed at 175 °C and the maximum value of 0.58 MPa at 130 °C. Overall, the results indicate that the proposed compensation algorithm exhibits high output accuracy and stability across the tested temperature range. However, a relatively larger RMSE is observed at 130 °C, suggesting that the nonlinear drift has not been completely eliminated, which may be attributed to local variations in the piezoresistive coefficient.

In addition, the maximum variation in sensitivity temperature drift is −0.019%FS/°C, showing a slightly nonlinear trend with temperature. This behavior indicates that the coupling effect between temperature-induced changes in the piezoresistive coefficient and diaphragm stress remains the main factor affecting sensitivity stability. Overall, the compensated output error is significantly reduced, confirming the effectiveness of the proposed model under high-temperature and high-pressure conditions.

## 4. Conclusions

This study addresses the development requirements of intelligent pressure sensors for ultra-deep drilling systems and investigates the design and implementation of a sensor output compensation method. To mitigate the temperature-induced drift in the output of downhole MEMS piezoresistive pressure sensors, a comprehensive software-based compensation strategy and an implementation framework are proposed. In particular, a temperature compensation algorithm based on the Modified Slime Mould Algorithm–BackPropagation (MSMA-BP) neural network is developed for high-temperature and high-pressure conditions, aiming to enhance measurement accuracy and thermal stability across the full-scale range of the sensor. The main conclusions are as follows:

1. Improved optimization algorithm: To overcome the limitations of the conventional Slime Mould Algorithm (SMA)—including insufficient search capability, susceptibility to local optima, and difficulty escaping stagnation—a Modified Slime Mould Algorithm (MSMA) was developed. The MSMA integrates chaotic initialization, an elite learning mechanism, and an adaptive variable-step search factor. Based on this improvement, an MSMA-BP temperature correction model was established, significantly enhancing the algorithm’s global optimization ability and convergence stability.
2. Integrated temperature–pressure compensation: A temperature–pressure coupled compensation model was established, and calibration experiments were conducted under various temperature and pressure conditions. Comparative analyses with several representative compensation algorithms demonstrate that the proposed MSMA-BP model achieves superior compensation accuracy and output consistency, effectively suppressing temperature-induced drift errors in the sensor output.
3. Embedded implementation: The proposed MSMA-BP model was deployed on an embedded hardware platform, achieving high-accuracy compensation while maintaining real-time performance and engineering feasibility.

The proposed compensation algorithm is not only applicable to improving the output accuracy of resistive pressure sensors but also holds potential for extension to other types of sensors or sensitive circuits. The high-temperature and high-pressure tests conducted in the laboratory provided controlled and repeatable data, establishing a solid foundation for systematically analyzing the temperature drift characteristics of the sensors and training the compensation model.

However, several limitations should be acknowledged. First, real-world downhole environments are far more complex than laboratory conditions, as sensors are subject to temperature gradients, geological pressure fluctuations, and drill tool vibrations, all of which introduce dynamic characteristics into their outputs. Additionally, slight nonlinear drift may still occur in the high-temperature range. Second, all experimental data were collected from sensors of the same manufacturing batch, and the total number of samples is relatively limited. Therefore, the generalization capability of the model to sensors from other batches or different fabrication processes has not yet been validated, and a larger dataset would help to better capture the nonlinear characteristics of the sensors, reduce the risk of overfitting, and enhance model stability. Third, inherent hardware characteristics of the sensors—including resolution, repeatability, and hysteresis—may influence measurement accuracy, and these parameters were not directly compensated for in the current study, representing an additional limitation. Although the compensation model is primarily built upon the physical response characteristics of sensors rather than batch-specific idiosyncrasies—and thus holds theoretical potential for cross-batch applicability—this premise requires experimental verification. Future research will focus on three main directions: (1) collecting data from sensors of multiple batches with a larger sample size to systematically evaluate cross-batch generalization performance; (2) exploring the applicability of the proposed method to other sensor types; (3) incorporating inherent hardware characteristics, such as resolution, repeatability, and hysteresis, into the compensation model for joint optimization; and (4) combining physical mechanism modeling with deep learning algorithms to further enhance the model’s generalization ability and stability in high-temperature and high-pressure environments.

## Figures and Tables

**Table 1:** Performance comparison of different temperature compensation methods.

Calibration Algorithm	Temperature Range	Pressure Range	Accuracy	Computing Budget
Look-up table	-	-	-	Low
interpolation	−20 °C–80 °C	−40 kPa–40 kPa	0.03%FS	Medium
Curvilinear fitting	−40 °C–40 °C	0–200 kPa	0.02%FS	Medium
Neural networks (BP)	20 °C–55 °C	0–10 kPa	0.48%FS	High
LSSVM	−10 °C–70 °C	−40 kPa–40 kPa	0.012%FS	High
Swarm Optimization algorithm	−20 °C–100 °C	0–100 kPa	0.002%FS	High
ELM	−40 °C–85 °C	0–20 MPa	2.57%FS	High
ASIC-MLP	25 °C–80 °C	0–80 kPa	0.5%FS	High
Passive	0–60 °C	0–60 psi	-	High

**Table 2:** Parameter table for benchmarking functions.

	Dim	Function	Expression	Range	Theoretical Min
F1	30	Sphere	$\sum_{i=1}^{n}x_{i}^{2}$	[−100, 100]	0
F2	30	Schwefel 2.22	$\sum_{i=1}^{n}x_{i}+\prod_{i=1}^{n}\left|x_{i}\right|$	[−10, 10]	0
F3	2	Easom	$-\cos(x_{1})\cos(x_{2})\times \exp[-{\left(x_{1}-\pi \right)}^{2}-{\left(x_{2}-\pi \right)}^{2}]$	[−100, 100]	−1
F4	30	Schwefel 2.21	$max\left\{\begin{array}{c} \left|x_{i}\right|,1\leq i\leq n \end{array}\right\}$	[−100, 100]	0
F5	30	Quartic	$\sum_{i=1}^{n}ix_{i}^{4}+rand[0,1)$	[−30, 30]	0
F6	2	Bridge	$\frac{\sin\sqrt{x_{1}^{2}+x_{2}^{2}}}{\sqrt{x_{1}^{2}+x_{2}^{2}}}-0.7129+\exp(\frac{\cos2\pi x_{1}+\cos2\pi x_{2}}{2})$	[−1.5, 1.5]	−0.56
F7	30	Generalized Rosenbrock	$\sum_{i=1}^{n}[100{\left(x_{i+1}-x_{i}^{2}\right)}^{2}+{\left(x_{i}-1\right)}^{2}]$	[−1.28, 1.28]	0
F8	30	Schwefel 2.26	$\sum_{i=1}^{n}-x_{i}\sin(\sqrt{|x_{i}|})$	[−500, 500]	−12,569.5
F9	30	Levy 1.3	$\begin{array}{l} 0.1\{\sin^{2}(\pi 3x_{1})+\sum_{i=1}^{29}{\left(x_{i}-1\right)}^{2}[1+\sin^{2}(3\pi x_{i+1})]\}\\ +{\left(x_{n}-1\right)}^{2}[1+\sin^{2}(2\pi x_{30})]+\sum_{i=1}^{30}u(x_{i},5,100,4) \end{array}$	[−50, 50]	0
F10	30	Ackley	$20+e-20\exp(-0.2\sqrt{\frac{1}{n}\sum_{i=1}^{n}x_{i}^{2}})-\exp(\frac{1}{n}\sum_{i=1}^{n}\cos(2\pi x_{i}))$	[−32, 32]	0

**Table 3:** Performance Comparison of MSMA Ablation Variants on Benchmark Functions.

	F2		F3		F4	
	Mean	Std	Mean	Std	Mean	Std
M0	0.00	0.00	3.14	1.14 × 10^−4^	0.00	0.00
M1	0.00	0.00	−1.00	8.37 × 10^−9^	0.00	0.00
M2	2.25 × 10^−159^	1.12 × 10^−158^	−1.00	1.97 × 10^−7^	3.24 × 10^−135^	1.78 × 10^−134^
M3	0.00	0.00	0.00	0.00	0.00	0.00
M4	0.00	0.00	−1.00	8.88 × 10^−8^	0.00	0.00
M5	0.00	0.00	0.00	0.00	0.00	0.00
M6	0.00	0.00	−1.00	9.43 × 10^−8^	0.00	0.00
M7	0.00	0.00	−1.00	1.01 × 10^−8^	0.00	0.00
	F5		F6		F7	
	Mean	Std	Mean	Std	Mean	Std
M0	−1.58 × 10^−2^	1.60 × 10^−2^	−2.07	2.31	8.22 × 10^−1^	3.35 × 10^−1^
M1	0.00	0.00	−5.61 × 10^−1^	1.86 × 10^−3^	0.00	1.08 × 10^1^
M2	1.95 × 10^−4^	2.23 × 10^−4^	−5.62 × 10^−1^	1.34 × 10^−3^	6.04	1.01 × 10^1^
M3	1.39 × 10^−4^	1.15 × 10^−4^	0.00	0.00	0.00	0.00
M4	1.72 × 10^−4^	1.81 × 10^−4^	−5.62 × 10^−1^	9.82 × 10^−4^	6.05	1.08 × 10^1^
M5	0.00	0.00	0.00	0.00	0.00	0.00
M6	1.72 × 10^−4^	1.37 × 10^−4^	−5.61 × 10^−1^	1.80 × 10^−3^	5.91	1.07 × 10^1^
M7	1.67 × 10^−4^	1.32 × 10^−4^	−5.62 × 10^−1^	1.34 × 10^−3^	7.67	1.17 × 10^1^
	F8		F9		F10	
	Mean	Std	Mean	Std	Mean	Std
M0	4.20 × 10^2^	2.59 × 10^−1^	9.72 × 10^−1^	3.84 × 10^−2^	−5.90 × 10^−16^	4.19 × 10^−16^
M1	−1.26 × 10^4^	1.68 × 10^−1^	2.19 × 10^−6^	1.69 × 10^−3^	0.00	0.00
M2	−1.26 × 10^4^	3.33 × 10^−1^	2.05 × 10^−3^	1.79 × 10^−3^	4.44 × 10^−16^	0.00
M3	0.00	0.00	0.00	0.00	0.00	0.00
M4	−1.26 × 10^4^	1.82 × 10^−1^	1.73 × 10^−3^	1.91 × 10^−3^	4.44 × 10^−16^	0.00
M5	0.00	0.00	0.00	0.00	0.00	0.00
M6	−1.26 × 10^4^	2.27 × 10^−1^	1.63 × 10^−3^	1.61 × 10^−3^	4.44 × 10^−16^	0.00
M7	−1.26 × 10^4^	2.55 × 10^−1^	1.70 × 10^−3^	1.45 × 10^−3^	4.44 × 10^−16^	0.00

**Table 4:** Main components of the test bench and their information.

Equipment	Model	Parameter
pressure pumps	SYB-250	Displacement: 15 mL/stroke, Max pressure: 230 MPa,
Temperature transducer	LHE6150A	Range: 0–175 °C
Pressure transducer	LHE6150D-09a	Range: 0–220 MPa, Repeatability Error: ±0.1% F.S, Hysteresis Error: ±0.15% F.S, Resolution: 0.4 MPa
DEES	LHE-DEES1.0	Win 11, RAM 4G, Database: Structured storage; Interface: USB 3.0
Control/Communication Fixture	LHE6154.221a	RS485, Baud Rate: 9600 bps, Data Bits: 8, Stop Bits: 1, Parity: None
DC power	GP60D03	Range: 1–150.0 V, Max current 3A

**Table 5:** BP Neural Network Structure and MSMA Parameters.

Category	Parameter	Description/Unit	Value
BP Neural network	Architecture (input–hidden–output)	Number of neurons in each layer	(2, 10, 1)
	Number of layers	Number of trainable weights and biases	3
	Learning rate	-	0.001
	Epochs	-	200
MSMA	Optimization dimension	Optimization variables (correspond to 41 BP parameters)	41
	Parameter bounds	Lower bound/Upper bound	(−30, 30)
	Population size	-	200
	Max iterations	-	200

**Table 6:** Calibration Results for Each Compensation Method.

Method	Max FS Error (%)	Avg FS Error (%)
Interpolation	1.07	0.27
Polynomial Interpolation	1.64	0.35
ELM	1.81	0.52
RELM	0.92	0.32
LSSVM	1.22	0.35
XGBoost	2.38	0.39
GR	0.76	0.31
BP	1.13	0.31
SMA-BP	0.59	0.17
MSMA-BP	0.51	0.15

**Table 7:** Performance Comparison of Various Temperature Compensation Methods.

Method	Max FS Error (%), Mean ± Std (Best)	Avg FS Error (%), Mean ± Std (Best)	Training Time	Testing Time
Interpolation	5.3 ± 3.8 (0.29)	0.54 ± 0.45 (0.08)	42.98 ms	2.06 ms
Polynomial Interpolation	1.27 ± 0.70 (0.49)	0.41 ± 0.20 (0.13)	16.9 ms	7.5 ms
ELM	0.95 ± 0.35 (0.56)	0.33 ± 0.07 (0.22)	117 ms	34.32 ms
RELM	3.71 ± 2.33 (1.11)	1.36 ± 0.76 (0.52)	126.1 ms	29.5 ms
LSSVM	1.22 ± 0.56 (0.59)	0.32 ± 0.09 (0.16)	48 ms	30 ms
XGboost	2.38 ± 1.78 (0.57)	0.39 ± 0.25 (0.15)	0.4 s	16.3 ms
GR	0.76 ± 0.40 (0.31)	0.31 ± 0.10 (0.10)	17.7 ms	2.7 ms
BP	1.15 ± 1.04 (0.36)	0.30 ± 0.16 (0.11)	4.18 s	168 ms
SMA-BP	1.10 ± 0.48 (0.41)	0.34 ± 0.15 (0.13)	7.16 s	111 ms
MSMA-BP	0.74 ± 0.23 (0.50)	0.26 ± 0.03 (0.20)	6.59 s	85.5 ms

**Table 8:** Non-correction point model prediction situation (part).

Sample	1	2	3	4	5	6	7	8	9	10
Input	115.247	118.925	59.797	94.650	7.7659	142.163	54.992	99.341	27.563	144.935
Predicted	115.248	118.937	59.74	94.673	7.7753	142.107	55.018	99.377	27.598	144.900
Abs error	0.001	0.011	0.057	0.023	0.009	0.055	0.026	0.035	0.034	0.035

**Table 9:** Pressure Compensation Verification Results.

*T*/°C	Pressure/MPa						
	0	20	60	100	140	160	170
25	0	19.9	60	100.3	140.2	/	/
60	0	20	59.6	99.6	139.4	/	/
100	0	19.8	59.6	99.6	139.7	/	/
130	0	20	60	100.3	138.8	161	171.1
175	0	20	60	100.1	140.1	160.4	170.4

## Data Availability

Derived data supporting the findings of this study are available from the corresponding author on request.
